# Oxidation modification of chitosan-based mesoporous carbon by soft template method and the adsorption and release properties of hydroxycamptothecin

**DOI:** 10.1038/s41598-020-72933-4

**Published:** 2020-09-25

**Authors:** Xianshu Wang, Qian Lin, Hongyan Pan, Shuangzhu Jia, Hong Wu, Yongyong Shi, Zhuhua Wang

**Affiliations:** 1 School of Chemistry and Chemical Engineering, Guizhou University, Guiyang, 550025 Guizhou People’s Republic of China; 2 School of Pharmacy, Guizhou University of Tradtinal Chinese Medicine, Guiyang, 550025 Guizhou People’s Republic of China; 3 Nano-Drug Technology Research Center, Guizhou University of Traditional Chinese Medicine, Guiyang, 550025 Guizhou People’s Republic of China

**Keywords:** Materials science, Nanoscience and technology

## Abstract

Spray drying and a direct carbonization technology were coupled to prepare nitrogen-doped mesoporous carbon nanoparticles (NMCs) using chitosan as a carbon source and nitrogen source precursor and a triblock amphiphilic copolymer (F127) as a soft template, then oxidative modification was performed by ammonium persulfate (APS) to prepare oxidized mesoporous carbon nanoparticles (O-NMCs). The pore structure, chemical composition and wettability of the mesoporous materials were studied before and after oxidative modification, the microscopic morphology, structure, composition and wetting performance of the mesoporous carbon were characterized by transmission electron microscopy (TEM), an X-ray diffractometer (XRD), N_2_ adsorption–desorption instrument, X-ray photoelectron spectroscopy (XPS), contact angle tests and other analyses, meanwhile influences of the mesoporous carbon material on adsorption and release performance of a poorly-soluble antitumor drug hydroxycamptothecin (HCPT) were investigated. It was demonstrated from results that the surface wettability of the oxidatively-modified mesoporous carbon material was improved, the contact angle of the mesoporous carbon materials was reduced from 133.4° to 58.2° and the saturated adsorption capacity of HCPT was 676.97 mg/g and 647.20 mg/g respectively. The dissolution rate of the raw material hydroxycamptothecin was improved due to the nanopore structure of the mesoporous carbon material, the dissolution rate of mesoporous carbon material-loaded hydroxycamptothecin was increased from 22.7% to respective 83.40% and 81.11%.

## Introduction

Mesoporous carbon materials possess a good application prospect in biomedicine due to their advantages of high specific surface area, large pore volume, adjustable pore size and easy surface modification. Hard template method^1–4^ and soft template method^5–8^ were commonly applied to the preparation of mesoporous carbon materials, Soft template method has advantages of simpler synthesis steps and lower cost compared with hard template method. However, now the phenolic resin synthesized from phenol and formaldehyde was mostly used as carbon source in the soft template method, which cannot meet the requirements of an environment-friendly mesoporous carbon preparation process without harmful residues of biological materials fundamentally. In addition, the carbonization process uses high temperature carbonization to remove the template, resulting in poor hydrophilicity of carbon materials and restricting its application in drug carriers. Studies showed that functionalization of mesoporous carbon materials by doping nitrogen atoms can greatly improve the wettability, biocompatibility and electrical conductivity of materials^9^, thus broadening its application in various fields.

C and N contents were 67.82 wt% and 9.65 wt%^10^ in chitosan as a cellulose-like and nitrogen-containing biopolymer^11^, with an abundant reserve, low price, renewable, good biocompatibility and environmental friendliness, in recent years, synthesis of mesoporous carbon materials increased gradually through chitosan as carbon source precursor. For example, Andrzej et al.^12^ and Peng et al.^13^ used chitosan as carbon source and colloidal SiO_2_ as template to prepare mesoporous carbon. Removal of silicon by HF enabled the synthesis steps complicated. Sun et al.^14^and Feng et al.^15^ prepared mesoporous carbon using chitosan as carbon and Pluronic triblock amphiphilic copolymer (F127) as template by solvent volatilized induced self-assembly (EISA). The mesoporous carbon prepared by the above method exhibits irregular morphology and particle size greater than 1 μm. In our previous study^16^, spherical mesoporous carbon materials were prepared by spray drying technology using chitosan as carbon and nitrogen source and F127 as template^16^. the prepared mesoporous carbon material had particle size less than 1 μm, a pore size of 3–6 nm, specific surface area of 274 ~ 868 m^2^/g, However, as the template is directly carbonized at 900 ℃, the oxygen-containing groups on the surface of carbon materials are reduced. Although the contact angle of 124.1° is lower than that of 161.9° of non-chitosan-based carbon materials^17^, the improvement of hydrophilicity is still not desired.

**Figure 1:**
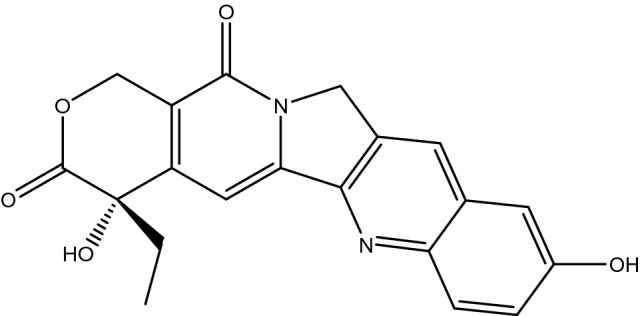
Molecular structure of hydroxycamptothecin.

In order to further improve the hydrophilicity of the mesoporous carbon surface and effectively address the issue of poor wettability and dispersion of the mesoporous carbon material as drug carrier, In this paper, nitrogen doped mesoporous carbon nanoparticles (NMCs) were prepared using chitosan as carbon and nitrogen source and F127 as template by the soft template method with the coupling of spray drying and carbonization process. Ammonium persulfate (APS) was employed to modify the mesoporous carbon materials, the nitrogen and oxygen co-doped modification was realized to improve the hydrophilicity of the carbon materials. The structural, surface chemical and hydrophilic properties of mesoporous carbon materials before and after modification were studied. Hydroxycamptothecin (HCPT, Fig. 1; Its molecular sizes was calculated by Materials Studio software as 1.14 nm × 0.69 nm × 0.44 nm) an insoluble antitumor drug was used as the model drug. The adsorption and release performance of HCPT by the mesoporous carbon materials were investigated before and after modification. It is beneficial to design a better mesoporous carbon carrier for drug delivery system, finally the dissolution rate and bioavailability of insoluble drugs were improved.

## Experiments

### Raw materials

Pluronic F127 (M_w_ = 12,600, EO_106_-PO_70_-EO_106_, American company Sigma-Aldrich), chitosan (degree of deacetylation ≥ 95%, viscosity of 100–200 mpa s, Aladdin reagent company), glacial acetic acid, anhydrous ethanol, Twen-80, potassium dihydrogen phosphate, sodium dihydrogen phosphate, sodium hydroxide, hydrochloric acid, Sodium bicarbonate (analytically pure, Chemical Reagent Co., Ltd. of Shanghai Sinopharm Group), ammonium persulfate (analytically pure, Tianjin Kermel Chemical Reagent Co., Ltd.), barium nitrate (analytically pure, Chengdu Jinshan Chemical Reagent Co., Ltd.), hydroxycamptothecin (HCPT-160201, Chengdu Yuancheng Biological Technology Co., Ltd.), deionized water as laboratory water.

### Preparation of nitrogen-doped mesoporous carbon (NMCs) through a chitosan-based soft template

The preparation process contained the following steps: 6 g of chitosan was dissolved in an aqueous solution of 5% acetic acid at 40 ℃ to prepare a 2% chitosan solution, 2 g of Pluronic F127 was dissolved in 100 ml pure ethanol containing Pluronic F127 at 40 ℃, the ethanol solution was added in chitosan solution through magnetic stirring at 40 ℃ for 60 min, which was then cooled down to room temperature for 24 h followed by drying the mixed solution through a spray dryer (BUCHI B-290, BUCHI company, Switzerland), a powder sample was prepared at an inlet air temperature of 170 ℃ with a material feeding flow rate of 3.5 ml/min, the obtained powder was put in a tube furnace with the raised temperature to 410 ℃ in an atmosphere of nitrogen at a rate of 2 ℃/min, thermal insulation was kept for 2 h, then the temperature was increased to 900 ℃ at a rate of 5 ℃/min, and continue to roasting at 900 ℃ for 2 h so as to obtain the nitrogen-doped mesoporous carbon material NMCs.

### Oxidative modification of O-NMCs

Oxidative modification included the following steps: 50 ml of 1 M ammonium persulfate solution ( the ammonium persulfate solution was prepared from 2.0 M H_2_SO_4_) was added in 0.5 g of the prepared nitrogen-doped mesoporous carbon (NMCs), which was stirred in a flask of 100 ml for reflux treatment at 70 ℃ with 8 h, then filtration was conducted followed by the washing with ethanol and water alternately until no precipitation was achieved through addition of barium nitrate into the filtrate, the filtered product was put into an oven at 100 ℃ with drying for 6 h so as to obtain the obtained sample marked as O-NMCs.

### Characterization method

Crystallization of the mesoporous carbon material was characterized by a D8 Advance-type X-ray diffractometer of German Bruker. The test conditions were as follows: Cu-target Kα radiation, incident wavelength λ = 0.154060 nm, a small angle range of 2θ = 0.5°–5°, a scanning angle range of 2θ = 5°–80°, a voltage of 40.0 kV, a current of 40.0 mA, a scanning speed of 2°/min and a scanning step length of 0.002°.

The microscopic morphology of the mesoporous carbon sample was characterized and analyzed by a Japanese FEI Tecnai G^2^ F20 S-Twin transmission electron microscope under the condition of field emission and a test voltage of 200 kV.

The specific surface area, pore volume and pore size of the mesoporous carbon were performed using an ASAP2020 N_2_ adsorption/desorption physical adsorption instrument of American Micrometrics. Sample treatment included the step of beforehand degassing under the vacuum condition of 76 mmHg at 120 ℃ for 12 h, the specific surface area (S_BET_) was calculated by a Barrett-Emmer-Teller method, and the pore volume (V_BJH_) and pore size (D_BJH_) were calculated using a Barrett–Joyner–Halanda (BJH) model of the isothermal adsorption branch, wherein the pore volume was obtained from the adsorption amount at the position with relative pressure P/P_0_ = 0.975.

The elemental composition (C, N and O) and atomic bonding state of the mesoporous carbon materials were characterized by a Thermo Scienftic Escalab 250XI-type X-ray electron spectrometer of the United States. Experimental conditions were as follows: an Al Kalph radiation source, test energy of 1486.8 eV, tested spot diameter of 500 microns, test tube voltage of 15 kV, tube current of 10 mA and analysis chamber background vacuum of 2 × 10^–9^ mbar, and C1*s* 284.8 eV was adopted as a standard for correction of peak positions during calibration.

The structural changes in the oxidized mesoporous carbon and drug-loaded mesoporous carbon samples were studied by Thermo Fisher Nicolet IS50 Fourier transform infrared spectrometer (FTIR) Experimental conditions: The powder samples were mixed with KBr, the wave number range was 4000–400 cm^−1^, and the instrument resolution was set to 16 cm^−1^.

The wettability of the mesoporous carbon was examined by an OCA25 video optical contact angle instrument of German Dataphysics.

#### Boehm titration

Modified Boehm titration method^18,19^ was used to determine the amount of oxygenated surface functional groups. In a 100 ml Erlenmeyer flask, 0.1 g of each sample was placed with 25 ml of the following solutions: 0.1 mol/l NaOH, 0.05 mol/l Na_2_CO_3_ , 0.1 mol/l NaHCO_3_ and 0.1 mol/l HCl. The flasks were shaken at 150 rpm for 24 h at 25 ℃.and then 5 ml of each solution was titrated with 0.05 mol/l HCl, except for the ones with 0.1 mol/l HCl which were back titrated adding first 10 ml of 0.1 mol/l NaOH. The amount of acidic sites of various types was calculated assuming that NaOH neutralizes carboxy, phenol and lactone groups; Na_2_CO_3_ neutralizes carboxy and lactone groups; and NaHCO_3_ neutralizes only carboxy groups. The number of basic sites was calculated from the amount of HCl that reacted with the sample.

### Adsorption of hydroxycamptothecin by mesoporous carbon

A standard curve of hydroxycamptothecin HCPT was obtained by the steps: 10 mg of hydroxycamptothecin HCPT was dissolved in 50 ml pure ethanol solution to prepare a standard stock solution of 200 μg/ml, the HCPT standard stock solutions were measured separately and precisely, 10 ml volumetric flasks was employed to prepare solutions with concentrations of 0.4, 0.5, 1, 3, 5, 7 and 10 μg/ml respectively, the pure ethanol solution was adopted as a reference solution to measure the absorbance values of the standard solutions of various concentrations at a maximum absorption wavelength of 385 nm by an ultraviolet spectrophotometer, regression analysis was carried out between the mass concentration (C) and the absorbance values (A) to obtain a regression equation: y = 0.07573x + 0.04149, wherein the standard curve had a good linear relationship between the absorbance values and the concentrations in the examined range of 0.4–10 μg/ml, the correlation coefficient R_2_ was 0.99947.

An HCPT adsorption experiment of the mesoporous carbon included the steps: accurately-weighed HCPT in absolute ethanol was dissolved to prepare a series of HCPT solutions with concentrations of 0.2–1.2 μg/ml, 20 mg of the mesoporous carbon carrier NMCs (O-NMCs) was added separately, which was then stirred in the dark at 37 ℃ for 24 h, then centrifugation was performed to examine the supernatants by an ultraviolet absorption spectrometer, the HCPT-loaded carbon material was put in a vacuum oven followed by drying at 40 ℃ for 24 h, and the HCPT-loaded NMCs and HCPT-loaded O-NMCs were marked as HCPT@NMCs and HCPT@O-NMCs. The drug adsorption amount of the mesoporous carbon materials was calculated based on the HCPT mass concentration values before and after adsorption, and the calculation formula was:

$$ q = V(C_{0} - C_{e} )/m $$

where in q was the unit adsorption amount (mg/g), C_0_ was the initial mass concentration (μg/ml) of the HCPT standard solutions, C_e_ denoted the mass concentration (μg/ml) of the HCPT solutions after adsorption, V represented the volumes (ml) of the HCPT standard solutions, and m was the mass (g) of the mesoporous carbon material sample.

### Drug release experiment of hydroxycamptothecin

The experiment included the steps: the dissolution amount of 15 mg of a crude drug HCPT and HCPT@NMCs and HCPT@O-NMCs with the corresponding drug loading were determined by dynamic dialysis, 0.1% Twen-80-containing phosphate buffer solutions (PBS) of pH 7.4 and 5.0 separately was selected as release mediums, then each sample was dispersed using 2 ml of the release mediums, which was put in pre-treated dialysis bags (MWCO = 14,000) with 500 ml of the release mediums, dissolution was performed in the dark in a dissolution apparatus at 37 ℃ at a rate of 100 r/min, taking 4 ml of the samples regularly at intervals of 1, 2, 4, 6, 8, 10 and 12 h, performing supplementation with fresh, isothermal and equal-volume phosphate buffers, taking out the dialysate with centrifugation at 2000 r/min for 10 min followed by dilution of 1 ml the supernatant 20 times, the absorbance value of each sample was measured three times using an ultraviolet spectrophotometer to calculate the concentration of the drug according to the standard curve, the accumulative release amount of hydroxycamptothecin was achieved as follows:

$$ Q\left( \% \right) = \frac{{V_{1} C_{n} + V_{2} \sum {C_{n - 1} } }}{W} \times 100\% $$

wherein, V_2_ was the sampling volume (ml), V_1_ was the medium volume (ml), C_n_ represented the release concentration (μg/ml) of hydroxycamptothecin at the nth sampling, n was the sampling times, and W was the drug content of hydroxycamptothecin in the mesoporous carbon material.

## Results and discussion

### Structural characteristics of mesoporous carbon materials

**Figure 2:**
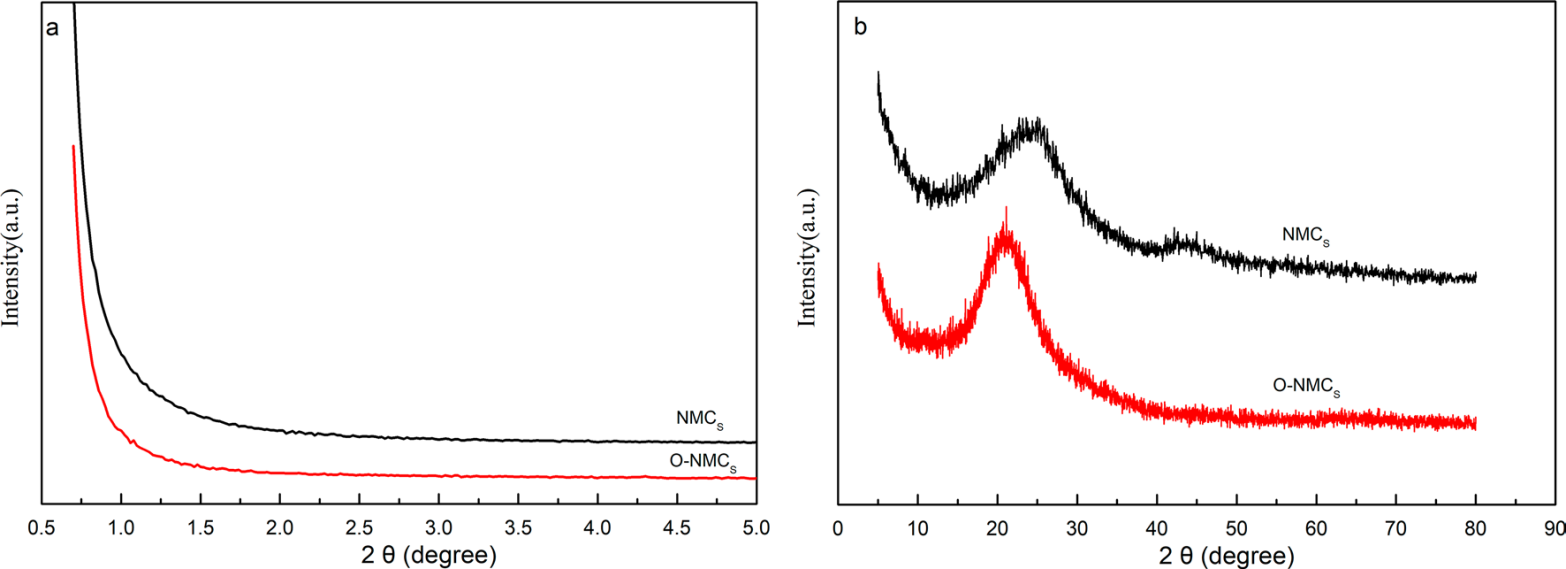
Low-angle (**a**) and wide-angle (**b**) XRD patterns of the pristine NMCs and oxidized O-NMCs.

The low angle XRD patterns of the pristine material NMCs and oxidized O- NMCs at 2θ of 0.7°–5° were shown in the Fig. 2a. No distinct diffraction peaks of pristine chitosan—based mesoporous carbon by the soft template method were observed, indicating that the material has disordered structures, Similar results were obtained for the samples oxidized O-NMCs with APS solution.

The XRD spectra of the NMCs and O-NMCs samples at 2θ of 5°–80° were shown in the Fig. 2b. Broad peaks at 2θ of 23° were observed in both the mesoporous carbon materials, which were attributed to amorphous peaks of carbon materials, it indicated that the carbon materials had an amorphous structure with the shift of broad peaks at 2θ of 23° to the left upon oxidation, implying that structural shrinkage occurred during oxidation. The sample NMCs had a small peak at 2θ of 43° before oxidation treatment, however it can be noted that the peak disappeared after oxidation, indicating that the structure of the mesoporous carbon was changed slightly after oxidative modification through ammonium persulfate.

**Figure 3:**
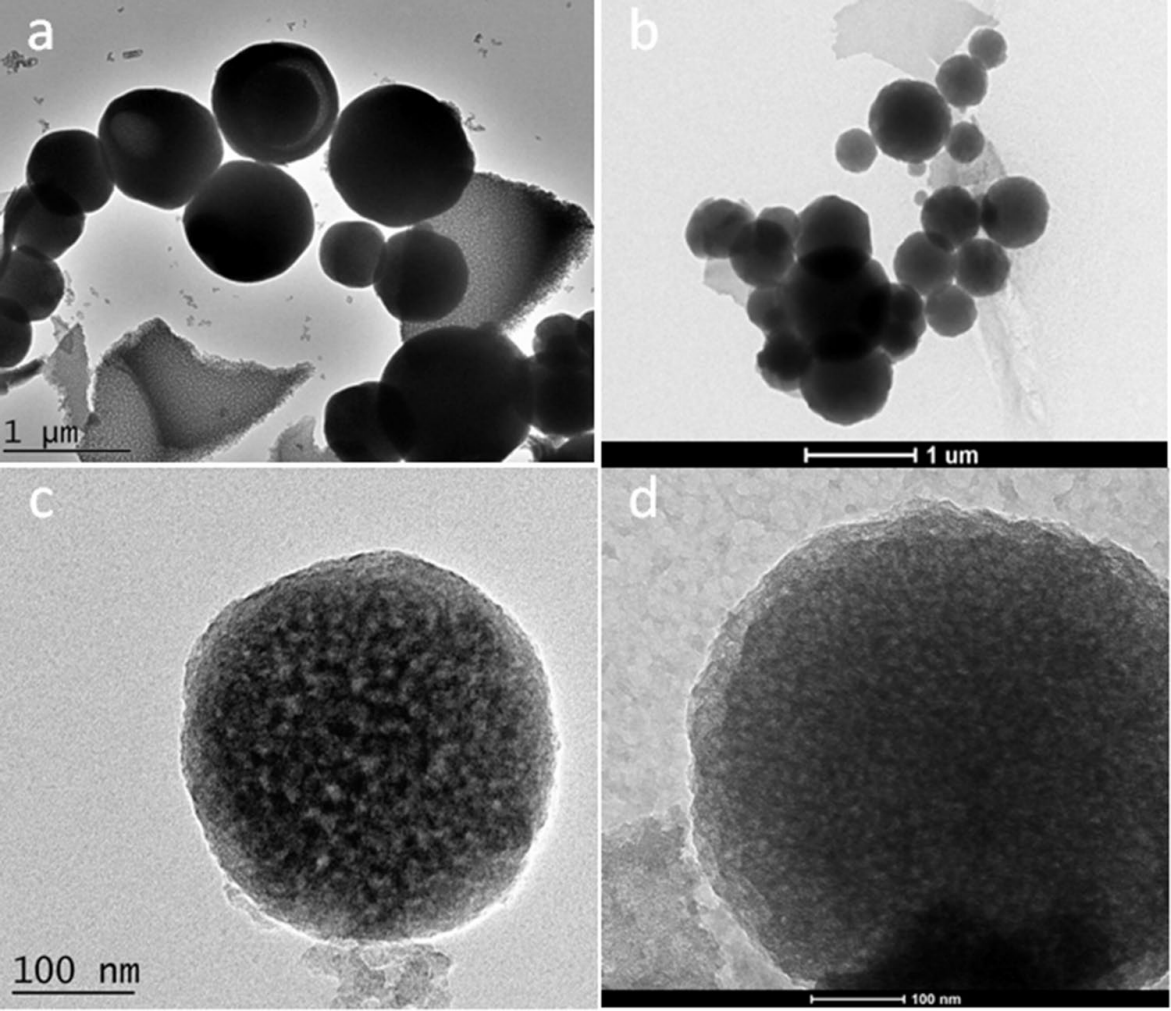
TEM images of the NMCs (**a**,**c**) and O-NMCs (**b**,**d**).

TEM images of the samples NMCs (a, c) and O-NMCs (b, d) were shown in the Fig. 3. It can be noted from the Fig. 3a and b that the mesoporous carbon NMCs prepared by the chitosan-based soft template method and the oxidized O-NMCs samples all had regular spherical morphology. It was shown in the Fig. 3c that the particle size of NMCs was about 200 nm. Figure 3c and d showed that the pores of the prepared mesoporous carbon were clear with a typical worm-like structure. The results consistent with the results of chitosan-based mesoporous carbon reported by Peng et al.^13^, as well as with the results of the XRD in the Fig. 2, the TEM images demonstrated that morphology and structure of the mesoporous carbon materials did not change distinctly upon oxidatively modification through ammonium persulfate.

**Figure 4:**
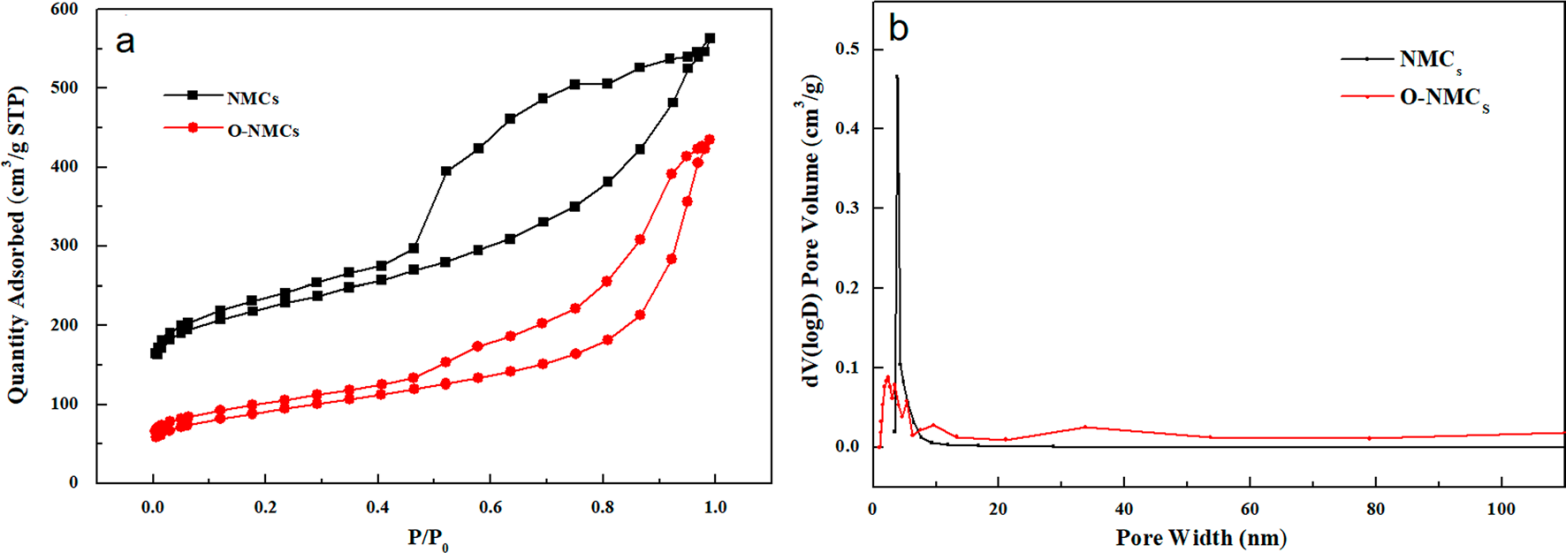
N_2_ adsorption–desorption isotherms and pore size distribution curves of the NMCs and O-NMCs.

The nitrogen adsorption–desorption isotherms and pore size distribution curve of the samples NMCs and O-NMCs were shown in the Fig. 4, the corresponding pore structure data was listed in the Table 1. As shown in the Fig. 4a, the nitrogen adsorption isotherms of both samples had hysteresis loops after relative pressure P/P_0_ at least 0.4, indicating that the NMCs and O-NMCs samples were both typical mesoporous materials, wherein the hysteresis loop of the sample NMCs was larger, and the corresponding mesopore pore volume was larger, the hysteresis loop of the sample O-NMCs was smaller, and the corresponding mesopore pore volume was smaller. It can be noted from the pore size distribution curve in the Fig. 4b that the pore size distribution of the mesoporous carbon materials was narrow upon modification with mainly concentrated around 2–4 nm. Table 1 shows that the average pore size of the oxidatively-modified mesoporous carbon was reduced from 3.99 to 1.80 nm, implying that the structure of the mesoporous carbon shrunk during the oxidation process, which was consistent with the XRD results. Prior to modification, the NMCs sample had a specific surface area of 804 m^2^/g and a pore volume of 0.87 cm^3^/g, the specific surface area of the oxidatively-modified mesoporous carbon O-NMCs was decreased to 322 m^2^/g, the pore volume was decreased to 0.64 cm^3^/g. It was demonstrated that the specific surface area and pore volume of the oxidatively-modified mesoporous carbon were reduced to a certain extent due to occupation of the original pore space by surface-modified functional groups of the oxidatively-modified mesoporous carbon, The reduction of specific surface area and pore volume after wet oxidation modification was described elsewhere^20,21^.

**Table 1:** Textural parameters of the NMCs and O-NMCs.

Sample name	S_BET_(m^2^/g)	V_meso_ (cm^3^/g)	D_me_(nm)
NMCs	804	0.87	3.99
O-NMCs	322	0.64	1.80

### Composition analysis of mesoporous carbon materials

The amount of surface functional groups through Boehm titration and elemental analysis was shown in Table 2. The amount of total acidic functional groups increased from an initial amount of 1.93 mmol/g for the pristine NMCs to 4.21 mmol/g for the oxidized sample O-NMCs when oxidant solution of APS was used. Especially, the increased carboxylic (–COOH) and phenolic (–OH) groups was favored, this was consistent with the results reported by Aguilar et al.^19^ for using APS oxidation solutions for activated carbon oxidation. The concentration of carboxylic groups and phenolic groups exhibited a similar tendency to that of the total acidic groups, however, the concentrations of lactonic groups was reduced.

**Table 2:** Amount of surface functional groups determined by Boehm titration and elemental composition of the mesoporous carbon materials NMCs and O-NMCs by XPS.

Sample	Surface functional groups (mmol/g)					Elemental composition (at.%)		
	Carboxylic	Lactonic	Phenolic	Total acidic	Basic	C	N	O
NMCs	0.55	1.28	0.10	1.93	0.64	91.66	4.59	3.750
O-NMCs	1.36	0.49	2.36	4.21	0.40	71.87	4.86	23.26

The increased oxygen of the samples after the oxidation process was also confirmed by elemental analysis data, as listed in Table 2. It was shown that the NMCs sample mainly contained C and a small amount of N and O elements. It was shown through XPS results that NMCs contained 91.66 at.% of C and 3.750 at.% of O, and the oxidized O-NMCs contained 71.87 at.% of C and 23.26 at.% of O upon wet oxidation through APS, indicating that the oxygen content of the oxidatively treated mesoporous carbon material O-NMCs was increased by about 19.51 at.%, meanwhile the number of oxygen-containing functional groups can be boosted up to 6.2 times through the wet oxidation method using APS due to the increased surface oxygen-containing functional groups after APS wet oxidation.These values are similar to those when using APS solutions to oxidize beaded activated carbon^19^.

**Figure 5:**
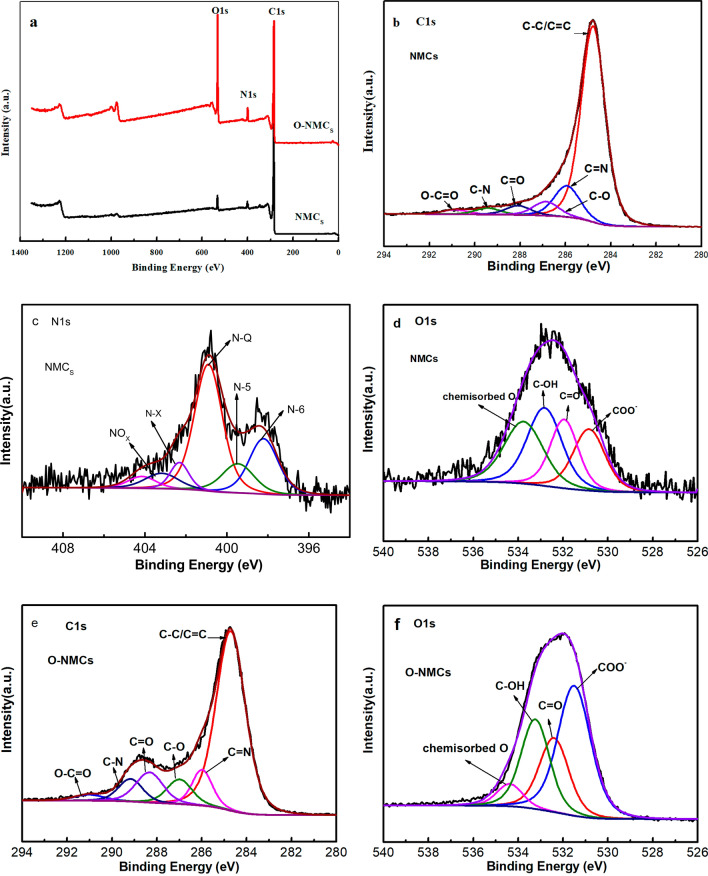
XPS spectra of of NMCs and O-NMCs: (**a**) XPS survey, (**b**,**e**) C1*s*, (**c**) N1*s* and (**d**,**f**) O1s.

XPS spectra of the NMCs and O-NMCs of the mesoporous carbon materials before and after modification were shown in Fig. 5. It was observed that the mesoporous carbon contained three elements of O, N and C before and after modification. Table 2 shows that the content of the C element in the mesoporous carbon material O-NMCs was reduced from 91.66 to 71.87%, and the content of the O element was increased from 3.75 to 23.26% upon oxidative modification. It was indicated that the introduced functional groups in the samples were mostly oxygen-containing groups after oxidation of the mesoporous carbon materials by ammonium persulfate, however the negligible change of content of the N element was achieved. The data of peak-differentiating and fitting of C1*s*, N1*s* and O1*s* were shown in Fig. 5b–f, which showing that the C1*s* in the mesoporous carbon material NMCs can be divided into five peaks, values of the corresponding binding energy were 284.6 eV, 285.8 eV, 287.7 eV, 288.5 eV and 289.3 eV respectively, corresponding to bonds of C=C, C=N, C=O, C–N and O–C=O^22^. A broad spectrum peak of C=O was observed in the C1s spectrum upon wet oxidation, indicating that more functional groups existed in the oxidatively-treated O-NMCs material due to the increased oxygen-containing groups of the oxidatively-modified mesoporous carbon material.

The N1*s* spectra of the NMCs was processed with peak differentiation and fitting (Fig. 5c), showing a splitting of N1*s* into five peaks with corresponding binding energies of 398.1, 399.2, 400.9, 402.1, and 403.20 eV respectively, which were attributed to pyridinic nitrogen (N-6), pyrrolic nitrogen (N-5), quaternary nitrogen (N-Q), Pyridinic-N-oxide (N-X) and nitrogen oxides (NO_X_)^11,23^, These results clearly indicated that in situ N was doped into the mesoporous carbons as the form of pyridinic, pyrrolic nitrogen and quaternary nitrogen.

The O1*s* spectra of the NMCs and O-NMCs were processed with peak differentiation and fitting (Fig. 5d, f), respectively. The O1*s* spectra of NMCs were divided into four peaks with corresponding binding energy of 531.6 eV, 532.7 eV, 533.8 eV and 534.3 eV respectively, which corresponded to the carboxyl groups COO–, C=O, C–OH and adsorbed chemisorbed O^22^. The peak values of the oxidatively-modified O-NMCs locating at 531.6 eV and 533.8 eV corresponded to the peaks of COO– and C–OH, this were much higher than those of the NMCs, indicating the formation of more oxygen-containing groups, wherein the hydrophilicity and wettability of the mesoporous carbon materials can be enhanced effectively through the carboxyl groups COO– and phenolic hydroxyl groups C–OH.

### Analysis of wettability of mesoporous carbon materials

**Figure 6:**
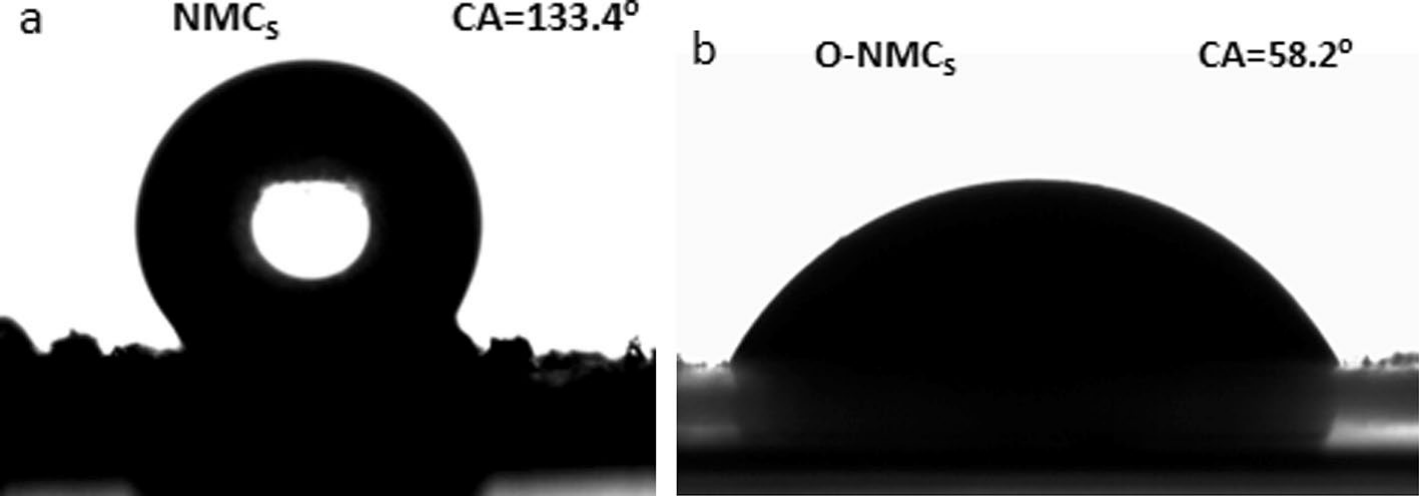
Photographs of water droplets on the surface of NMCs (**a**) and NMCs (**b**) and corresponding contact angles.

Pictures of water droplets on the surface of NMCs (a) and O-NMCs (b) and the corresponding contact angles were shown in the Fig. 6. It can be observed that the static contact angle of the mesoporous carbon NMCs was larger than 133.4° before oxidation, no spreading was observed from the spherical water droplets on the surface of the NMCs, demonstrating that the NMCs had hydrophobicity. The static contact angle of the oxidized mesoporous carbon material O-NMCs was reduced to 58.2°, the water drops spread well on the surface of the O-NMCs, implying the hydrophilicity for O-NMCs. The contact angle was much lower than 129° of mesoporous carbon materials prepared using phenolic resin as a carbon source^24^, and the reason was that the surface hydrophilicity of the ammonium sulfate oxidation-modified mesoporous carbon material was improved due to introduction of a large number of hydroxyl groups, carboxyl groups and other oxygen-containing functional groups on the surface of the mesoporous carbon material^25^. Therefore, the wettability of the mesoporous carbon materials can be improved significantly upon oxidative modification using ammonium persulfate, so that improvement of the contact opportunities between the aqueous solutions and the materials was facilitated, and the application range of the mesoporous carbon materials was expanded better.

### Study on hydroxycamptothecin adsorption and release performance of NMCs and O-NMCs

**Figure 7:**
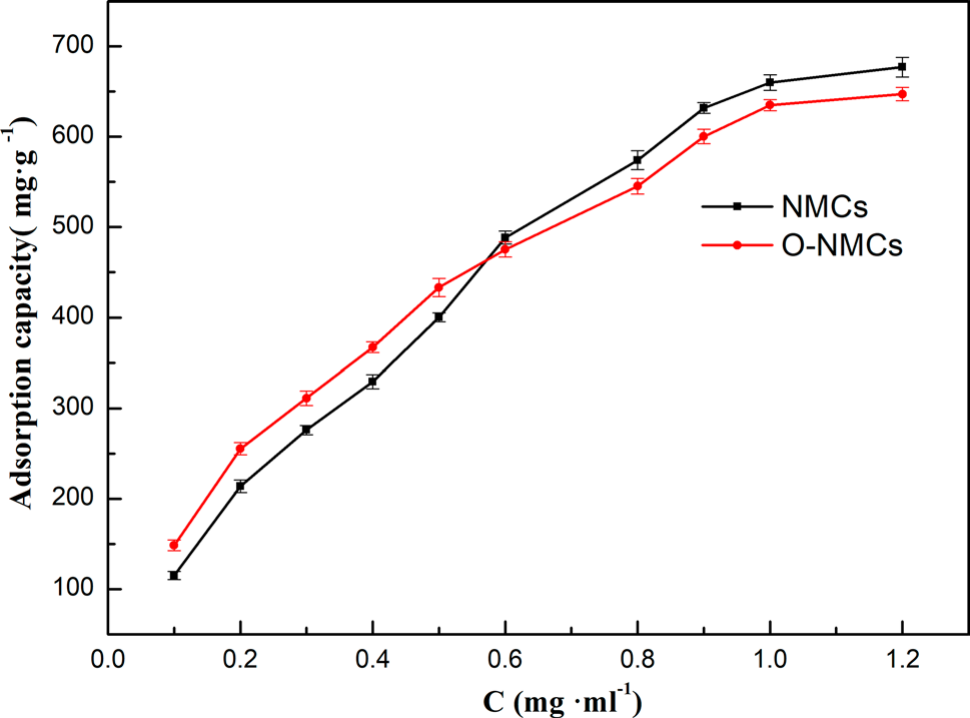
HCPT adsorption isotherms of NMC_S_ with O-NMCs in ethanol solution, Error bars were based on triplet samples.

The HCPT adsorption curves of NMCs and O-NMCS in the ethanol solutions were shown in Fig. 7. It can be noted that the HCPT adsorption capacity of the mesoporous carbon materials was enhanced gradually with the increased HCPT concentration in the solutions because adsorption of HCPT in the porous materials was diffusion adsorption mainly followed the concentration gradient principle. The higher HCPT concentration led to the stronger driving force of the concentration gradient. More HCPT entered the adsorption sites on the surface of the mesoporous carbon for adsorption and enrichment, as a result, the adsorption amount of HCPT became larger.

The Freundlich model was applied for the fitting of the experimental data in the figure, the data was shown in the Table 3.

**Table 3:** Freundlich constants of mesoporous carbons after drug loading with HCPT.

Sample name	K_F_( mg/g)	n	R^2^
NMCs	657.789	1.459	0.9850
O-NMCs	627.739	1.741	0.9904

The Freundlich adsorption formula was given as follows:

$$ q{\text{ } = \text{ K}}_{{\text{F}}} {\text{c}}^{\frac{1}{n}} $$

wherein q was the mass (mg/g) of adsorbed HCPT in carbon material pores of unit mass at equilibrium, K_F_ denoted the Freundlich adsorption equilibrium constant (mg/g), c was the concentration (mg/mL) of HCPT at adsorption equilibrium, and n was a constant related to the temperature and system.

It can be noted from the Fig. 6 and Table 3 that adsorption of the HCPT molecules in the pores of the carbon materials followed the Freundlichr adsorption law, and the value of the adsorption constant K did not change significantly, indicating similar HCPT affinity of the mesoporous carbon materials before and after oxidation. Noticeably, the saturated adsorption capacity of HCPT by the mesoporous carbon materials NMCs and O-NMCs was respective 676.97 mg/g and 647.20 mg/g at a drug loading of at least 40.37% which was much higher than the HCPT loading (24%) of three-dimensional macroporous carbon materials without nitrogen doping reported in the literature^26^. Furthermore, it was shown through the adsorption curve that HCPT adsorption capacity of O-NMCs was larger due to better wetting properties of O-NMCs compared with NMCs at low concentrations of HCPT. O-NMCs had a smaller specific surface area and a smaller pore volume than those of the NMCs mesoporous carbon with the increased concentration of HCPT, therefore the HCPT adsorption capacity was reduced at higher concentrations.

**Figure 8:**
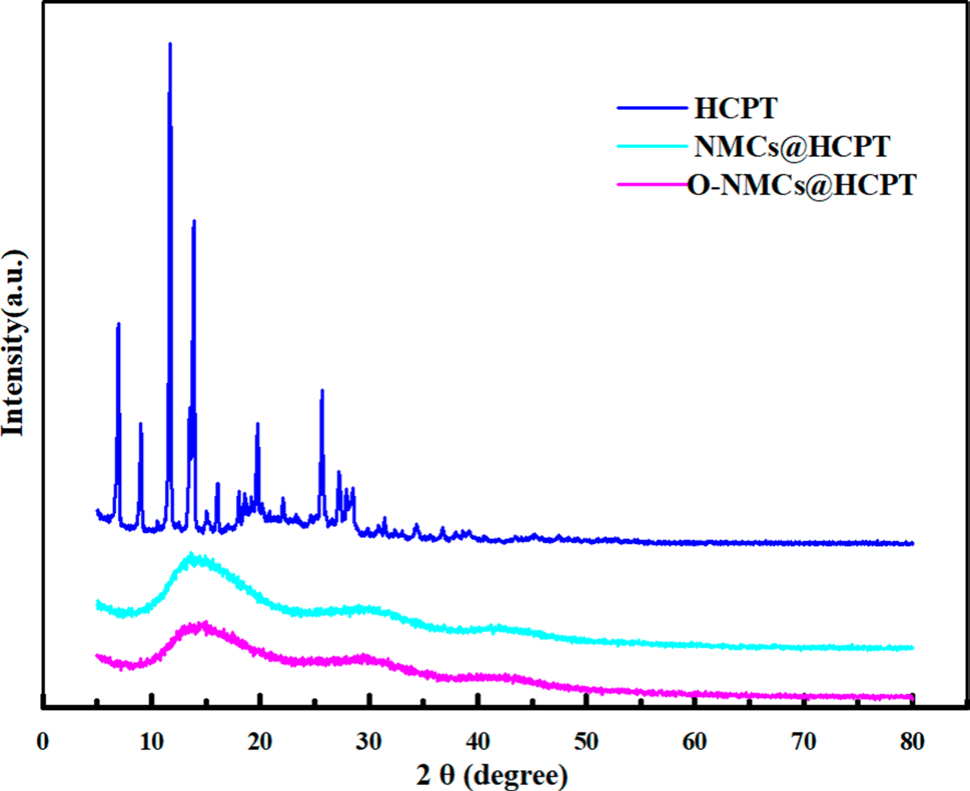
The XRD patterns of pure HCPT, NMCs@HCPTand O-NMCs@HCPT.

The X-ray diffraction results of HCPT, NMCs@HCPT and O-NMCs@HCPT were shown in Fig. 8. The crude drug HCPT had strong crystal diffraction peaks locating at 2θ of 6.9°, 9.0°, 11.70°, 13.86°, 19.73°, 25.65°, 27.27°, 27.91° and 28.52°, indicating the existence of the crude drug HCPT at a crystalline state. However, the diffraction peaks of HCPT cannot be observed in the NMCs@HCPT and O-NMCs@HCPT samples when the HCPT was loaded on the mesoporous carbon materials, indicating an amorphous state for the adsorbed HCPT in the mesoporous carbon carriers. The results were consistent with reports from Qinfu Zhao group^27^, the drug can be kept at an amorphous state through the nanopores of the mesoporous carbon, so that the dissolution rate of the drug was improved.

**Figure 9:**
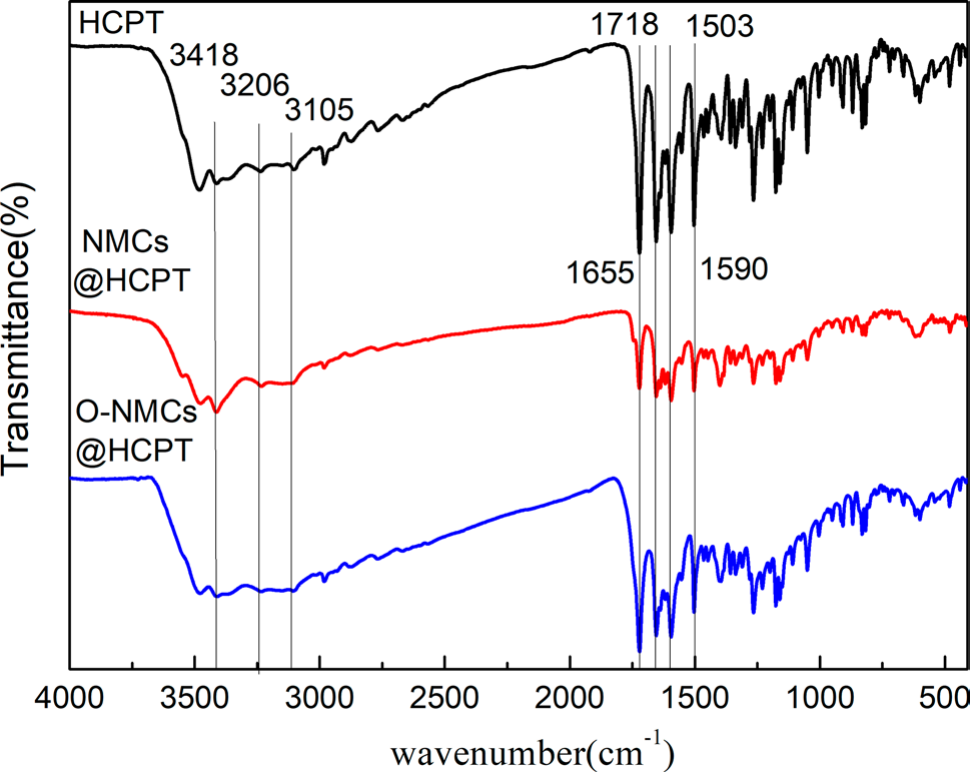
The FTIR spectra of pure HCPT, NMCs@HCPT and O-NMCs@HCPT.

The FTIR spectra of HCPT, NMCs@HCPT and O-NMCs@HCPT were shown in the Fig. 9. In the spectrum of HCPT, the characteristic peak at 3418 cm^−1^ was attributed to the stretching vibration of tertiary alcohol hydroxyl group, the bands at 3206 and 3105 cm^−1^ can be assigned to stretching vibration of phenolic hydroxyl, the stretching vibration of C=O in ester group was responsible for the absorption peak at 1718 cm^−1^, the characteristic band at 1655 cm^−1^ was ascribed to the acylamino group. Furthermore, the peaks at 1590 cm^−1^ and 1503 cm^−1^ were assigned to the absorption bands of the aromatic ring in HCPT. The characteristic bands of HCPT can be observed upon the loading of HCPT, confirming that HCPT molecules were indeed loaded into mesoporous carbon materials. The changed peak strength and a small shift of peak position indicated that the mesoporous carbon material and HCPT drug had a synergistic effect.

**Figure 10:**
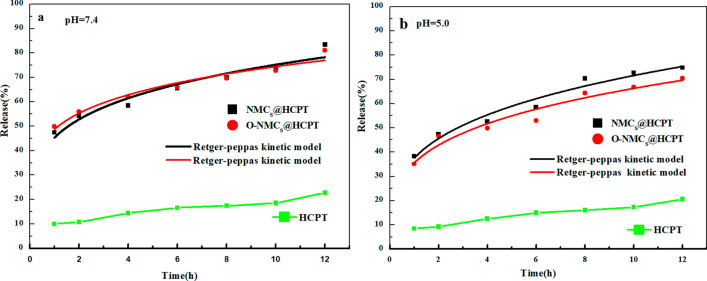
In vitro release profiles of HCPT, NMCs@HCPTand O-NMCs@HCPT in pH 7.4 (**a**) and pH 5.0 (**b**) PBS solution.

The in-vitro drug release behavior curves of HCPT, NMCs@HCPT and O-NMCs@HCPT in PBS solutions of pH values of 7.4 and 5.0 were shown in Fig. 10a and b respectively. It can be observed from the Fig. 10a that the release amount of HCPT in the PBS solution with pH 7.4 after 1 h was only 9.96%, only 22.7% of HCPT was released after 12 h. By contrast, the release rate of the HCPT drug molecules was improved greatly upon the adsorption of HCPT on NMCs and O-NMCs, 47.44% and 49.78% of the drug were released from NMCs@HCPT and O-NMCs@HCPT respectively after 1 h, meanwhile amount of drug release can boost up to 83.40% and 81.11% respectively after 12 h. It can be seen from the Fig. 10b that the release amount of HCPT in the PBS solution at pH 5.0 under acidic conditions for 1 h was only 8.44%, and only 12.56% was released after 12 h. By contrast, the release rate was also significantly higher than that of the crude drug HCPT after the adsorption of HCPT drug molecules on NMCs and O-NMCs, however 38.24% and 35.04% of the drug was released from the drug-loading materials NMCs and O-NMCs after 1 h under acidic conditions compared with a neutral environment at pH 7.4, and the drug release amount can reach up to 74.78% and 70.45% respectively after 12 h. It was shown in the paper that the release in an acidic environment of pH 5.0 was slower than that in a neutral environment of pH 7.4 after the HCPT drug was loaded on the mesoporous carbon, indicating that the release of HCPT from the mesoporous carbon materials was associated with the pH value. The release rate of HCPT was improved significantly compared with the crude drug HCPT after the HCPT drug was loaded on the mesoporous carbon. Because crystallization of the drug can be inhibited to a certain extent through a unique nanopore structure of the mesoporous carbon, as a result, the drug was absorbed on the mesoporous carbon at the microcrystalline or amorphous state, so that the solubility and release rate of the drug were improved^28^.

The Retger-peppas kinetic equation was applied for the fitting of the experimental data in the Fig. 10, the data was shown in the Table 4.

$$Q = {{kt}^{n}} $$

where in Q was the release rate of the HCPT drug, t denoted the time, and k and n represented the release rate constant and index, respectively.

**Table 4:** Parameters obtained by fitting of Retger-peppas kinetic equation.

Sample name	k	n	R^2^
**pH 7.4**			
HCPT@NMCS	48.69	0.219	0.931
HCPT@O-NMCs	45.37	0.184	0.947
**pH 5.0**			
HCPT@NMCS	37.51	0.280	0.965
HCPT@O-NMCs	35.52	0.270	0.946

It can be noted that the drug release rate K was closely related to the wettability of the mesoporous carbon materials. The O-NMCs with excellent hydrophilicity exhibited a lower release rate and a smaller K value, but the NMCs with poor hydrophilicity exhibited a higher release rate and a larger K value. It may be induced by the fact that the O-NMCs with good hydrophilicity had more active sites compared with the NMCs with poor hydrophilicity, thus possessing a strong interaction force on the HCPT drug, so that it was difficult for the drug to get rid of the carbon material and diffuse into the release mediums.

The release rate of the hydroxycamptothecin drug in the acidic environment of pH 5.0 was slower than that in the neutral environment of pH 7.4. Therefore, it can be noted that the release rate of hydroxycamptothecin depended on the pH value, implying that the release rate would be slower in an environment with a lower pH value. Since the microenvironments of the tumor extracellular tissue, the intracellular lysosome and endosome were acidic^29^, the purpose of long-term resistance to tumors can be achieved through slow release of HCPT in an acidic environment of a phosphate buffer solution at pH 5.0. Above all, the higher oxygen content of the mesoporous carbon material resulted in better hydrophilicity. But when the comparison between the oxidatively-modified mesoporous carbon material O-NMCs and the unmodified NMCs was conduced, the adsorption amount of the anti-tumor drug HCPT was reduced due to the increased specific surface area. The higher oxygen content on the mesoporous carbon material led to the better hydrophilicity, finally the drug release rate became slower. Therefore, the release rate of HCPT can be adjusted through the varied wetting properties or oxygen content and the pH value of the mesoporous carbon materials.

## Conclusion

In this paper, the hydrophilicity of the mesoporous carbon surface was regulated to address issues of poor wettability and dispersion of the mesoporous carbon material as a drug carrier. The oxygen-containing functional group was introduced into the mesoporous carbon surface to study the variations of the structural properties, surface chemical properties and hydrophilicity of the mesoporous carbon material before and after modification. The adsorption and release properties of hydroxycamptothecin before and after modification of mesoporous carbon materials were studied using the insoluble antitumor drug hydroxycamptothecin as a model drug. The results show as follows.

The nitrogen-doped mesoporous carbon material was successfully prepared using chitosan as the carbon and nitrogen source, the triblock copolymer F127 as the template agent and the coupled technology of spray drying and pyrolysis, the mesoporous pore diameter was mainly concentrated at 3.99 nm, and the specific surface area and pore volume were 804 m^2^/g and 0.87 cm^3^/g respectively. The specific surface area, pore volume and pore size of the O-NMCs were reduced upon oxidative modification through ammonium persulfate, but the oxygen content was increased remarkably due to more formation of hydroxyl groups, carboxyl groups and other oxygen-containing groups on the surface, meanwhile the contact angle was reduced from 133.4° to 58.2°, as a result, the wettability was improved significantly, the mesoporous carbon materials with hydrophobic properties became hydrophilic.

NMCs and O-NMCs exhibited good adsorption performance of the anti-tumor drug hydroxycamptothecin HCPT, which had loading of HCPT of 676.97 mg/g and 647.20 mg/g, respectively. The release rate of the crude drug HCPT was improved upon the load of the drug HCPT on the mesoporous carbon material, the release rate of the crude drug after 12 h was only 22.7%, and the release rate was increased by 83.40% after HCPT was loaded on the mesoporous carbon materials. It was demonstrated through researches that the release rate of the drug HCPT can be reduced through the O-NMCs with higher oxygen content due to more active sites of the O-NMCs, the release amount was larger in the neutral environment at pH 7.4 compared with that in the acidic environment at pH 5.0, therefore the release rate of HCPT can be adjusted by the varied oxygen content of the mesoporous carbon materials and pH values of the release mediums. The mesoporous carbon material O-NMCs possessed a good pore structure, nano-morphology and wettability, and has good application potential as a delivery carrier of the poorly-soluble anti-tumor drug.
